# Propofol Increases Host Susceptibility to Microbial Infection by Reducing Subpopulations of Mature Immune Effector Cells at Sites of Infection

**DOI:** 10.1371/journal.pone.0138043

**Published:** 2015-09-18

**Authors:** Lavanya Visvabharathy, Bobbi Xayarath, Guy Weinberg, Rebecca A. Shilling, Nancy E. Freitag

**Affiliations:** 1 Department of Microbiology and Immunology, University of Illinois at Chicago, Chicago, Illinois, United States of America; 2 Department of Anesthesiology, University of Illinois at Chicago, Chicago, Illinois, United States of America; 3 Department of Pulmonary, Critical Care, Sleep and Allergy, University of Illinois at Chicago, Chicago, Illinois, United States of America; East Carolina University School of Medicine, UNITED STATES

## Abstract

Anesthetics are known to modulate host immune responses, but separating the variables of surgery from anesthesia when analyzing hospital acquired infections is often difficult. Here, the bacterial pathogen *Listeria monocytogenes* (*Lm*) was used to assess the impact of the common anesthetic propofol on host susceptibility to infection. Brief sedation of mice with physiologically relevant concentrations of propofol increased bacterial burdens in target organs by more than 10,000-fold relative to infected control animals. The adverse effects of propofol sedation on immune clearance of *Lm* persisted after recovery from sedation, as animals given the drug remained susceptible to infection for days following anesthesia. In contrast to propofol, sedation with alternative anesthetics such as ketamine/xylazine or pentobarbital did not increase susceptibility to systemic *Lm* infection. Propofol altered systemic cytokine and chemokine expression during infection, and prevented effective bacterial clearance by inhibiting the recruitment and/or activity of immune effector cells at sites of infection. Propofol exposure induced a marked reduction in marginal zone macrophages in the spleens of *Lm* infected mice, resulting in bacterial dissemination into deep tissue. Propofol also significantly increased mouse kidney abscess formation following infection with the common nosocomial pathogen *Staphylococcus aureus*. Taken together, these data indicate that even brief exposure to propofol severely compromises host resistance to microbial infection for days after recovery from sedation.

## Introduction

Microbial infection is a major complication for surgical patients in the US, occurring in the aftermath of ~2% of all surgeries [1]. While over 99% of surgery patients receive prophylactic antibiotics, the incidence of post-operative infections remains high, adversely impacting patient outcomes and increasing healthcare costs by $1-$10 billion dollars per year [2]. Significant effort has been focused on reducing patient exposure to infectious agents at the time of surgery; recently attention has also been directed to determine whether anesthetics impact patient susceptibility to infection.

Propofol is the most common anesthetic induction agent used in surgery and for routine outpatient procedures, such as gastrointestinal endoscopy [3], and the drug has been reportedly associated with immunomodulatory effects *in vitro* [4]. In cell culture studies, propofol exposure has been linked with decreased secretion of pro-inflammatory immune signaling molecules such as TNF-alpha and decreased expression of inducible nitric oxide synthase (iNOS) as well as decreased macrophage phagocytosis [5]. Propofol suppresses the production of prostaglandin E(2) (PGE_2_) by dendritic cells [6] and macrophages [7,8], and PGE_2_ is known to suppress inflammatory cytokine production and to stimulate production of interleukin (IL)-10 [9]. A recent i*n vivo* study using a cecal ligation and puncture model of sepsis in rats has indicated that continuous exposure to propofol for 24 hours resulted in elevated mortality compared to rats exposed to inhalation anesthetic [10]. Given the potentially broad clinical implications of immune modulation by propofol, we investigated mechanisms underlying propofol’s influence on host innate immunity following brief periods of sedation using two well-characterized *in vivo* mouse models of bacterial infection. Our results indicate that propofol significantly enhances host susceptibility to pathogen infection by inhibiting the recruitment and/or activity of immune effector cells at sites of infection.

## Materials and Methods

### Ethics statement

This study was carried out in strict accordance with the recommendations in the Guide for the Care and Use of Laboratory Animals of the National Institutes of Health. The protocol was approved by the Animal Care Committee of the University of Illinois at Chicago (Approved protocol number 12–061). Female outbred Swiss Webster mice, 6–8 weeks of age (Charles River Laboratories, Chicago, IL) were subjected to a 12-hr light/dark cycle with free access to food and water. Animals were carefully monitored twice daily for signs of distress (ruffling of fur, signs of listlessness) and humane endpoints were used in all experiments such that any animal exhibiting severe discomfort, an inability to move around or attain food, water, etc., was euthanized immediately. Euthanasia was carried out via CO2 inhalation from a bottled source followed by cervical dislocation. Animal suffering and distress was minimized by monitoring the animals as described above and in that tail vein injections were carried out with only brief periods (< 5 minutes) of physical restraint.

### Bacterial strains, media, and culture conditions

Mouse intravenous infections with *Lm* were carried out with wild type 10403S (WT) or an *actA* deletion mutant (*ΔactA*) [11]. All strains were grown in BHI medium (Difco Laboratories, Detroit, MI). Intravenous *Staphylococcus aureus* infections were performed using the methicillin resistant USA300 strain (courtesy of Dr. Victor Torres and Dr. Francis Alonzo). USA300 was grown overnight in TSA broth (MP Biomedicals, Solon, OH).

### Drug types and formulations

Propofol (Abbot Labs, North Chicago, IL) was used at a concentration of 18.75 mg/kg, a dose comparable with that used to induce anesthesia in humans based on FDA guidelines accounting for differences in metabolism [12] and which resulted in approximately three to five minutes of sedation. Intralipid (Sigma Aldrich, St. Louis, MO) + 5% dextrose was used as the vehicle control. For *in vitro* experiments, propofol was used at a final concentration of 50 μM. Ketamine/xylazine and sodium pentobarbital were used at concentrations of 25 mg/kg, 4 mg/kg and 50 mg/kg respectively for mouse experiments. All drugs and intralipid carrier were administered intravenously via tail vein injection.

### Intravenous infections of mice

**Fig 1:**
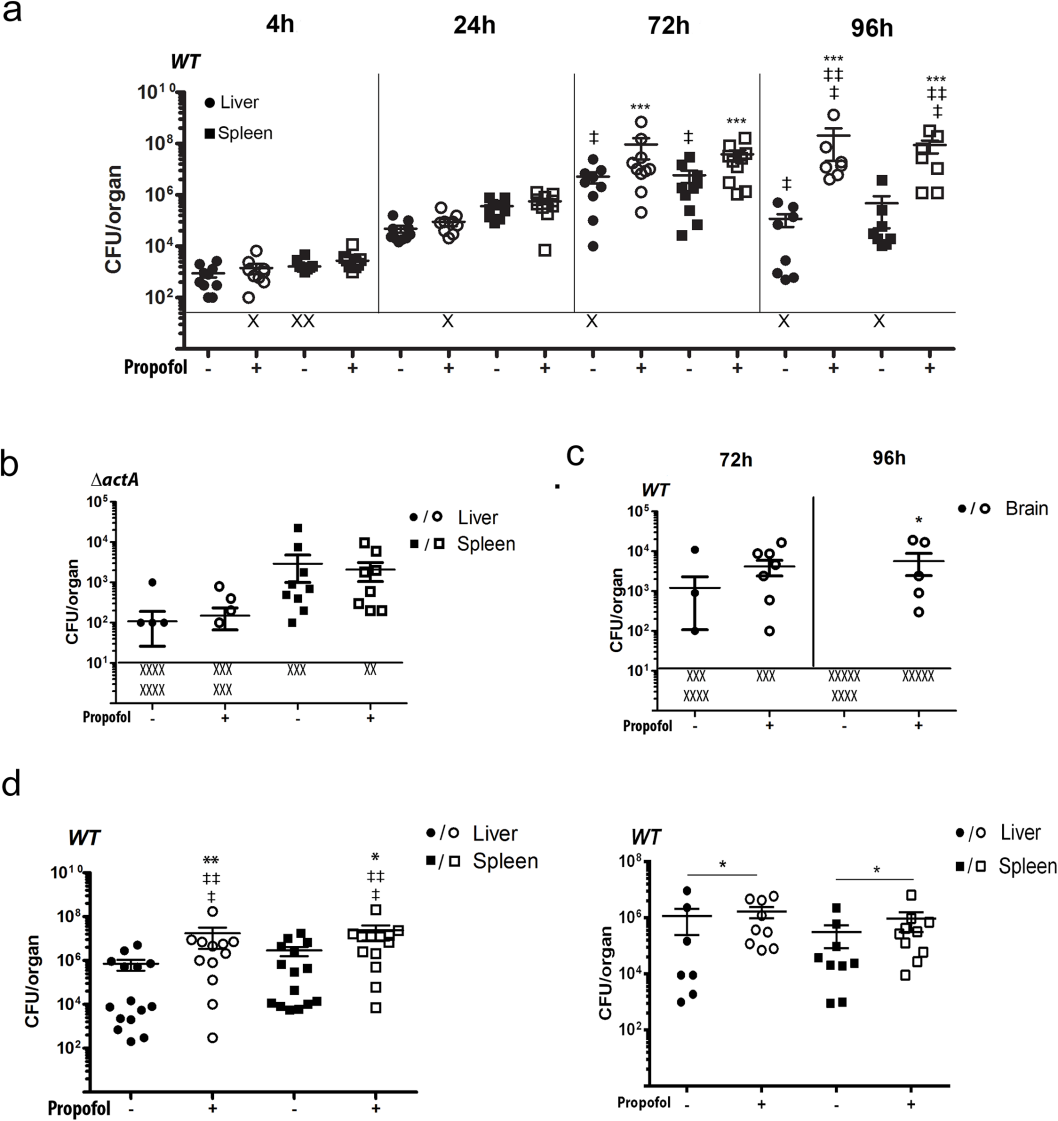
Propofol inhibits immune clearance of *Lm* from infected tissues. (a). Mice were intravenously infected with a sublethal dose of 2 x 10^4^ CFU *Lm* in the presence and absence of propofol exposure. Graph shows bacterial burdens present in target organs at the indicated time points post-infection. ‘X’ indicates animals with bacterial burdens that were below detection limits. Double dagger indicates animals that succumbed to infection prior to experimental endpoints. Data shown is from two independent experiments. (b). Propofol does not increase host susceptibility to *Lm* strains attenuated for virulence. Mice were intravenously infected with 2 x 10^4^ CFU *Lm ΔactA* (deficient for bacterial cell-to-cell spread) in the presence and absence of propofol exposure. Graph depicts bacterial burdens present in target organs at the indicated time points post-infection. ‘X’ indicates animals with bacterial burdens that were below detection limits. Data shown is from two independent experiments. (c). Propofol enhances *Lm* translocation across the blood-brain barrier. Mice were intravenously infected with 2 x 10^4^ CFU *Lm* in the presence and absence of propofol exposure. Graph depicts bacterial burdens present in the brain at the indicated time points. ‘X’ indicates animals with bacterial burdens that were below detection limits. Data shown is from two independent experiments. (d). The immunosuppressive effects of propofol are evident for up to four days post-sedation. Mice were intravenously infected with a low dose of 2 x 10^3^ CFU of *Lm* at 24 or 96 hours following brief propofol sedation. Bacterial burdens in the livers and spleens were determined at 96 hours post-infection. ‘X’ indicates animals with bacterial burdens that were below detection limits. Double dagger indicates animals that succumbed to infection prior to experimental endpoints. Data shown is representative of two independent experiments, error bars indicate data ± SEM. * p<0.05, ** p<0.005, *** p<0.001.

*Lm* overnight cultures were diluted and grown to mid-log phase in BHI broth. Bacteria were washed several times in sterile PBS and resuspended in PBS. Immediately prior to infection, 100 ul of bacterial suspension was mixed with 100 ul of vehicle solution (Intralipid + 5% dextrose) or propofol or pentobarbital suspension as indicated: this allowed delivery of both bacteria and drug through a single tail vein injection after determining that the suspension of *Lm* in the drug solutions did not affect bacterial viability for more than two hours post-suspension (Fig A in S1 File). Adolescent female Swiss Webster mice were infected via the tail vein with a sublethal dose of 2 x 10^4^ CFU or where indicated 2 x 10^3^ CFU plus drug or intralipid vehicle. For experiments using ketamine/xylazine, we determined that even brief exposure of *Lm* to these drugs was toxic and therefore mixing of the bacteria with drug immediately prior to tail vein injection was not possible. As we found that propofol exposure increased mouse susceptibility to infection for at least 4 days post recovery from sedation (Fig 1), to avoid any direct toxicity of ketamine/xylazine with injected *Lm* we first sedated the mice using tail vein injection with ketamine/xylazine and then tail vein injected *Lm* 24 hours after recovery from sedation. At indicated times, organs were harvested and homogenized and homogenates were used to determine viable CFU. *S*. *aureus* USA300 overnight cultures were diluted into fresh media and grown for ~3 hours. Cultures were washed with sterile PBS and diluted to the infectious dose of 1–3 x 10^6^ CFU/100 μl. Immediately prior to infection, bacteria were mixed 1:1 with vehicle or propofol solution. Organs were harvested at indicated times, homogenized, and viable CFU were determined.

### Cell culture infection assays

Bone marrow-derived macrophages (BMMs) and peritoneal macrophages were obtained from female 6–8 week old Swiss Webster mice as previously described [13]. Macrophages were placed onto glass coverslips overnight. Medium was supplemented with 50 uM propofol and/or 25 ng/ml of LPS for 2 hours and/or 1 ng/ml of IFN-γ (Biosource, Carlsbad, CA) for 18 hours prior to infection. Infections and bacterial CFU quantifications were performed as described [14].

### Bioplex cytokine and chemokine assays

3–5 mice were used per treatment group for all assays in two independent experiments. Mice were sacrificed at indicated times and whole blood was drawn via cardiac puncture. Serum was isolated and used in custom Bioplex cytokine assays (Bio-Rad Laboratories, Hercules, CA). Plates were read using the Bioplex 200 plate reader and analyzed with Bio-Plex Manager 5.0 software.

### IL-10 receptor blockade

Mice were infected with 1 x 10^4^ CFU of *Lm* intravenously via the tail vein in the presence or absence of propofol (vehicle controls received intralipid carrier). At 48 hours post-infection, they were given via tail vein injection 250μg of IL-10 receptor blocking antibody or 250μg of isotype control (both from BD Pharmingen, San Diego, CA). Animals were sacrificed at 72 hours post-infection, and livers and spleens were isolated and processed for enumeration of viable bacteria.

### Histological examination of infected tissues

Mice infected via the tail vein with 2 x 10^4^ CFU *Lm* or 1 x 10^6^ CFU *S*. *aureus* with propofol or with Intralipid carrier were euthanized at indicated times and spleens or kidneys were isolated. Organs were resuspended in 10% PBS-buffered formalin (Sigma-Aldrich, St. Louis, MO) overnight and prepared for histological analysis by the UIC Research Resource Center Histology Core and stained with hematoxylin and eosin (H&E).

### Flow cytometry

Mice were intravenously infected with 2 x 10^4^ CFU *Lm* in the presence and absence of propofol via the tail vein and euthanized at the indicated time points. Spleens were isolated and processed for flow cytometry as described by Pamer et al [15]. The following fluorescent and non-fluorescent primary antibodies were used: anti-Ly-6C (AL-21), Mac-3, CD11c (BD Pharmingen, San Diego, CA); anti-CCR2 (Abcam, Cambridge, MA), anti-NOS2 (C-11 epitope) (Santa Cruz Biotechnology, Santa Cruz, CA), anti-F4/80, 33D1, Ly-6G, CD-11b (Ebioscience, San Diego, CA), anti-TNF-α (clone MP6-XT22) (Biolegend, San Diego, CA). FACS was performed with a Cyan ADP flow cytometer, and data were analyzed with Summit software.

### Immunofluorescent staining

Paraffin-embedded organs were sectioned into 5μm sections and mounted onto glass slides by the UIC Research Resource Center Histology Core. Samples were then deparaffinized with xylene and rehydrated with graded ethanol washes. Antigen retrieval was performed using 10mM sodium citrate buffer in a pressure cooker at high pressure for 15 minutes. Slides were cooled and then incubated in a cold solution of 10% H_2_O_2_ to quench endogenous peroxidases and limit nonspecific red blood cell staining. Slides were then washed in Tris-buffered saline (TBS) and samples were marked with PAP pen circles. Background Buster blocking solution (Innovex Biosciences, Richmond, CA) was applied to each sample for 30 minutes prior to incubation in primary antibody. Samples were washed in TBS then incubated with rabbit antiserum against *Lm* (BD Pharmingen, San Diego, CA) and rat anti-mouse SIGN-RI antibody against marginal zone macrophages in the spleen (AbD Serotec, Raleigh, NC), or rabbit anti-mouse CD3 antibody against T cells (Abcam, Cambridge, MA) for 1 hour at room temperature or overnight at 4°C. Slides were washed in TBS then incubated sequentially with donkey anti-rabbit AF-488 or goat anti-rat AF-594 (Abcam, Cambridge, MA) for 1 hour at room temperature. Slides were once again washed in TBS and coverslips were mounted using ProlonGold Antifade Reagent with DAPI (Invitrogen, Grand Island, NY). Slides were imaged using the Zeiss Axio Imager A2 manual upright research microscope.

### Statistical analysis

All statistical analyses for *in vivo* infection assays were performed using a Mann-Whitney U test. ‘X’s representing undetectable bacterial burdens (less than 100 CFU) were input as 99 CFU in order to be conservative with statistical analyses. Statistical analyses for multiplex cytokine assays used an unpaired student’s t Test with Welch’s correction. All statistical analyses for flow cytometry were performed using one-way ANOVA with a Holm-Sidak multiple comparison test or two-way ANOVA with a Bonferroni multiple comparisons test. All statistical analyses for quantifying immunofluorescence data were performed using an unpaired two-tailed Student’s t Test, after first confirming that the data fell within a normal distribution using a Kolmogorov-Smirnov test.

## Results

### Mice exposed to propofol fail to clear bacteria from target organs

*Listeria monocytogenes* (*Lm*) is a facultative intracellular bacterial pathogen that has been used for decades as a tool to elucidate host immune responses to bacterial infection via mouse infection models [16–18]. We therefore examined the outcome of *Lm* infection in mice briefly sedated with propofol. Swiss Webster mice were given propofol intravenously at a dose of 18.75mg/kg via the tail vein, sufficient for less than 5 minutes of sedation and, importantly, comparable to induction doses in humans after correcting for differences in metabolic rates between humans and rodents [12]. Control animals were given intravenous Intralipid carrier, and both groups were intravenously inoculated via the tail vein with a sub-lethal dose (for Swiss Webster mice) of 2 x 10^4^ CFU of *Lm* mixed immediately prior to injection with either drug or Intralipid carrier. Bacterial burdens in drug treated and non-drug treated animals were similar up to 24 hours post-infection, however by 72 hours propofol-treated animals began to show significant increases in bacterial numbers within the liver and spleen (Fig 1A). These differences were more pronounced at 96 hours post-infection at which time bacterial numbers began to decrease in control animals while propofol-treated animals began to succumb to infection (Fig 1A). Propofol did not impair the ability of mice to control infection when exposed to attenuated *Lm* Δ*actA* strains, which are more than 1000-fold less virulent than wild type *Lm* [19] (Fig 1B), and there was no evidence that the drug enhanced bacterial replication within primary macrophages *in vitro* (Figure A in S1 File) despite reported drug effects on macrophage function [5]. Propofol sedation was additionally found to enhance bacterial translocation to the brain (Fig 1C and Figure B in S1 File). Propofol thus appears to increase host susceptibility to microbial infection via inhibition of immune responses that limit bacterial replication within tissues and impede blood-brain barrier translocation.

### Propofol exposure increases host susceptibility to infection up to 96 hours after recovery from sedation

To determine if the influence of propofol on host susceptibility to infection extended beyond sedation, mice were anesthetized with propofol or given Intralipid carrier via the tail vein and allowed to recover for 24 or 96 hours before intravenous tail vein infection with a low sublethal dose (2 x 10^3^ CFU) of *Lm*. Animals infected 24 hours after recovery from sedation still exhibited significantly increased bacterial burdens in target organs at 4 days post-infection in comparison to controls, with a number of anesthetized animals succumbing to infection (Fig 1D, left panel). Differences in bacterial burdens were detectable in animals infected 96 hours after propofol exposure (Fig 1D, right panel), and up to 7 days post-drug exposure (Figure C in S1 File), although the magnitude of the difference was much reduced.

### Alternative anesthetics do not recapitulate the effects of propofol on infection outcome

**Fig 2:**
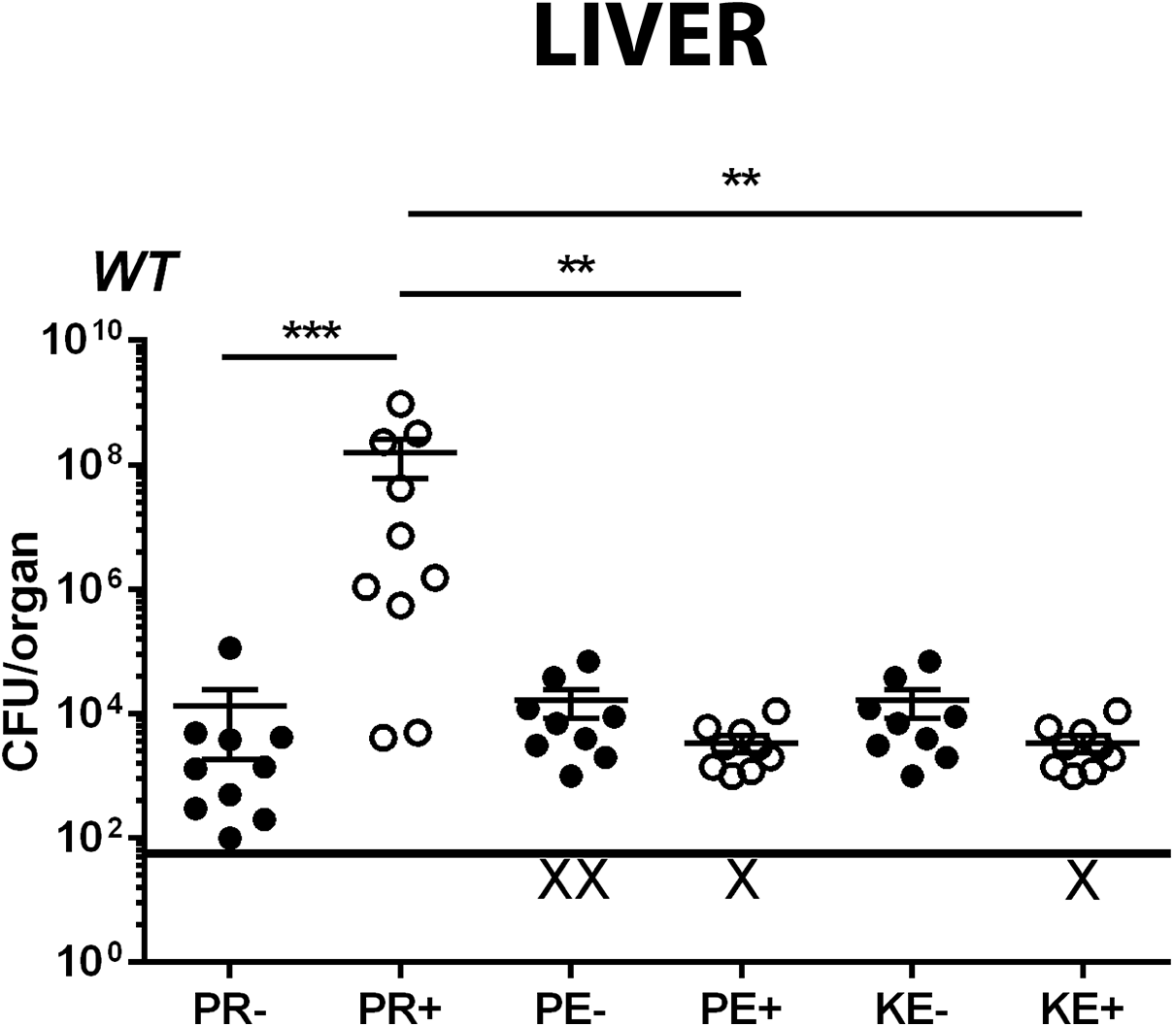
The anesthetics sodium pentobarbital and ketamine/xylazine do not increase host susceptibility to *Lm* intravenous infection. Mice were intravenously infected with a low sublethal dose of 2 x 10^3^ CFU *Lm* together with intravenous injection of vehicle solution, propofol, pentobarbital, or alternatively infection was carried out 24 hours after sedation with ketamine/xylazine to avoid direct toxic effects of the drug on bacteria. Graph depicts bacterial burdens in the liver at three days post-infection. ‘X’ indicates animals with bacterial burdens that were below detection limits. Data shown is combined from two independent experiments, error bars indicate data ± SEM. * p<0.05, *** p<0.0005

Pentobarbital and propofol target gamma amino-butyric acid A (GABA-A) receptors [20] [21,22], whereas the anesthetic ketamine binds to the N-methyl-D-aspartate (NMDA) receptor [23] and induces anesthesia via an alternate mechanism. GABA-A receptors are also present on macrophages and T cells, where they have been implicated in modulation of lymphocyte function and phagocytosis [24,25]. To determine if, similar to propofol, pentobarbital and ketamine reduced immune clearance of *Lm* from target organs, animals were intravenously infected with a sublethal low dose of *Lm* (2 x 10^3^ CFU) in the presence or absence of drug exposure. After 72 hours of infection, there was no significant difference in bacterial burdens recovered from target organs of pentobarbital or ketamine treated animals in comparison with vehicle-treated controls (Fig 2). This was in contrast to animals sedated with propofol, which exhibited significant increases in bacterial burdens in both liver (Fig 2) and spleen (data not shown). Immune suppression mediated by propofol does not therefore result from general sedation and is not mimicked by other anesthetics that target GABA-A receptors.

### Propofol alters the expression of host cytokines and chemokines during infection

**Fig 3:**
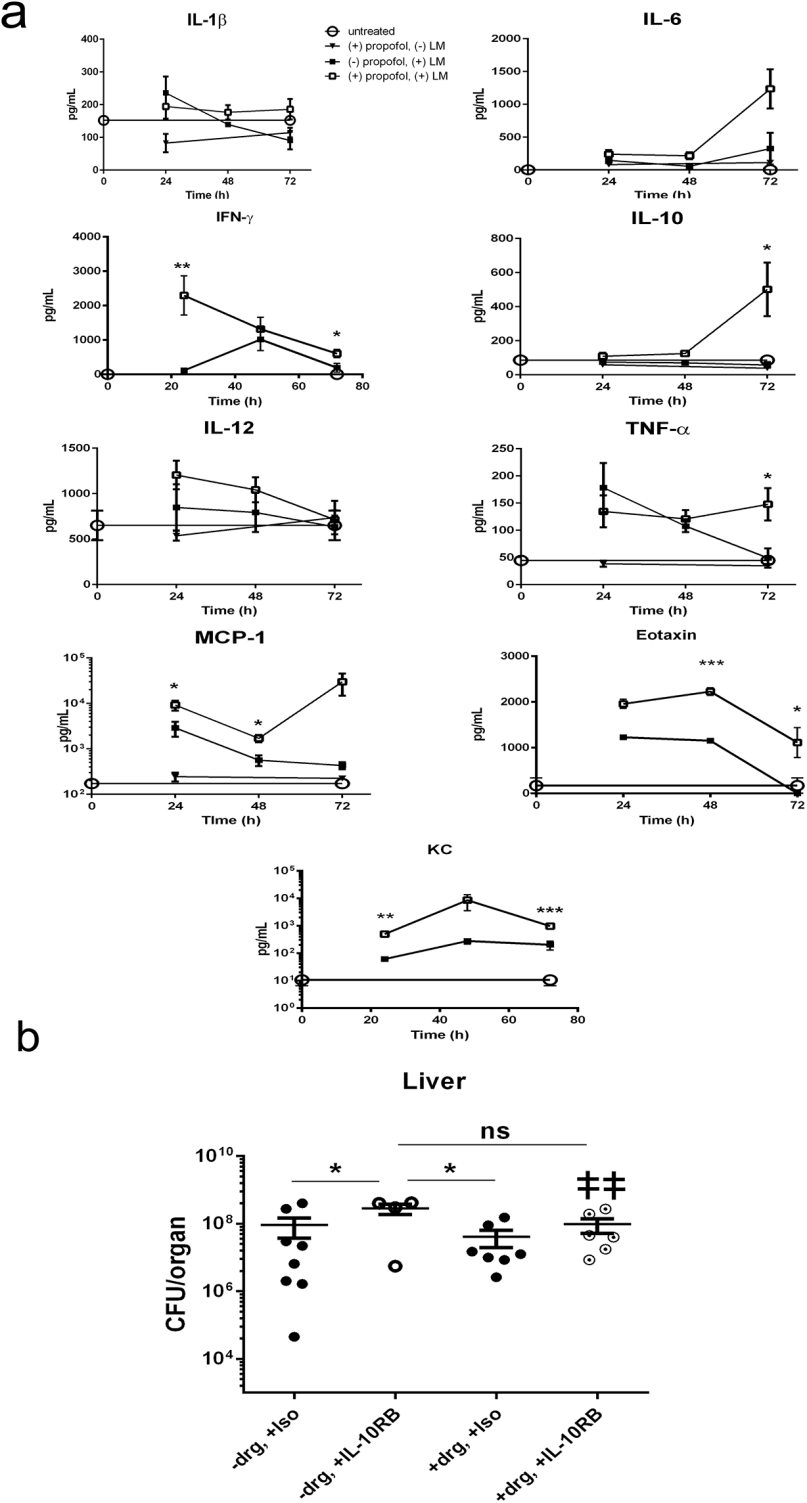
Propofol exposure alters host serum cytokine and chemokine expression following *Lm* infection. (a). Mice were intravenously infected with 2 x 10^4^ CFU of *Lm* in the presence and absence of propofol sedation. Serum samples were collected via cardiac puncture and analyzed for the presence of the indicated cytokines and chemokines. All statistical comparisons compare infected control animals with infected animals given propofol. (b). Mice were intravenously infected with 1 x 10^4^ CFU of *Lm* in the presence or absence of propofol. Mice were given the following treatments at 48 hours post-infection: antibody isotype control (-propofol and +propofol group), or 250μg of IL-10 receptor neutralizing antibody (-propofol and +propofol group). Graph depicts bacterial burdens in the liver at 72 hours post-infection; although differences between animals with and without propofol sedation are most visible at 96 hours post-infection with this inoculum, the experiment had to be terminated by 72 hours as propofol sedated animals began to succumb to infection. Data points represent 4–8 animals per treatment group per time point; double dagger indicates animals that succumbed prior to experimental endpoints, error bars indicate data ± SEM. * p<0.05, ** p<0.005, *** p<0.0001.

Given that propofol increased host susceptibility to *Lm* infection, we examined the expression patterns of key cytokines and chemokines associated with immunity to *Lm* [17]. Serum levels of the pro-inflammatory cytokines IL-1ß, IL-6, IL-12, and TNF-α and the anti-inflammatory IL-10 appeared similar in anesthetized and Intralipid control animals during the first 48 hours post-infection (Fig 3A). Levels of IL-1ß, IL-6, and TNF-α increased by 72 hours post-infection in drug treated animals, a time point at which bacterial burdens were just beginning to become significantly different than those of controls (Fig 1A). The chemokine MCP-1 (CCL2), which contributes to monocyte recruitment to sites of infection, was significantly higher at all time points in the propofol-treated groups, as were the eosinophil chemoattractant eotaxin and the neutrophil chemoattractant KC (CXCL1) (Fig 3A). IFN-γ, associated with resistance to *Lm* infection through the activation of Th1-type immune responses [26], was elevated in propofol-treated groups compared to controls at 24 hours post-infection, but decreased to control levels in animals at 48 and 72 hours post-infection (Fig 3A). In contrast, levels of IL-10 increased significantly at 72 hours post-infection in drug treated animals (Fig 3A). This raised the question as to whether the increase observed in IL-10 levels served to directly antagonize the production and activity of IFN-γ [27]. IL-10 receptor blockade via antibody treatment increased susceptibility of mice to *Lm* infection relative to infected mice given isotype control antibody alone or propofol plus isotype control (Fig 3B). A significant increase in bacterial burdens was observed for mice receiving IL-10 receptor blocking antibodies versus isotype control in the absence of sedation, and sedated mice subjected to IL-10 receptor blockade exhibited increased mortality, such that the experiment required termination at 72 hours post-infection instead of 96 hours post-infection (Fig 3B). These data indicate that under these conditions, increased IL-10 secretion enhanced host survival rather than compromised resistance to infection, potentially by limiting the damage caused by high level inflammation.

### Propofol exposure reduces immune effector cell populations at sites of infection

**Fig 4:**
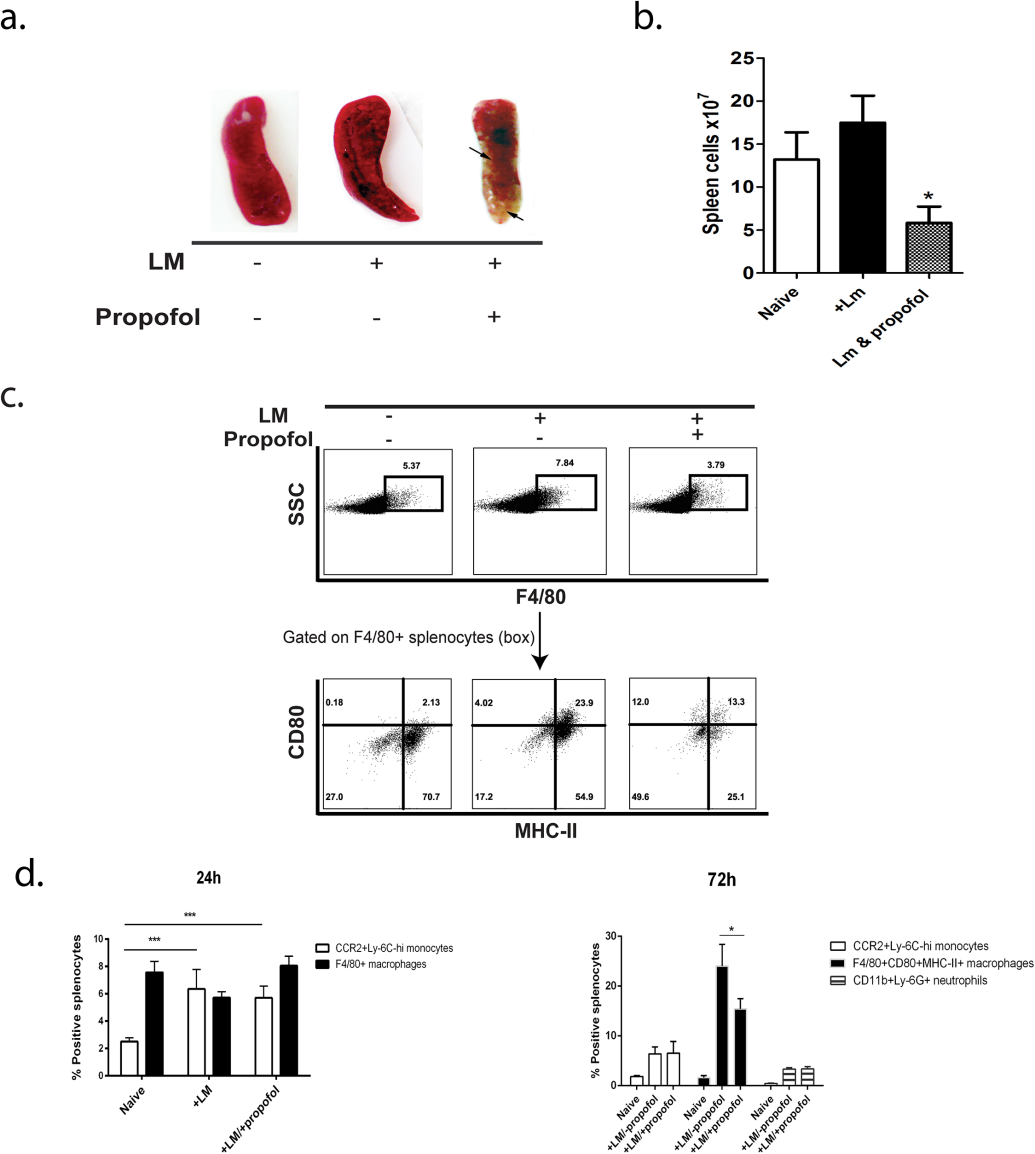
Propofol anesthesia results in loss of splenic architecture and reduced numbers of immune effector cell populations at sites of infection. (a). Spleens of anesthetized, infected animals were reduced in size and exhibited signs of necrosis at 72 hours post-infection. (b) Total splenocyte counts were lower in propofol-treated animals compared with infected controls. (c). Analysis of cell populations in the spleens of *Lm* infected animals ± propofol. Swiss Webster mice were left uninfected or infected i.v. with 2 x 10^4^ CFU of *Lm* in the presence or absence of propofol and spleens were processed for FACS. At 72 hours post-infection, anesthetized animals had proportionally fewer F4/80^+^CD80^+^MHC-II^+^ mononuclear phagocytes than infected controls. (d). At 24 hours post-infection, no significant differences in the proportional numbers of inflammatory monocytes (CCR2+Ly-6C^hi^) or F4/80^+^CD80^+^MHC-II^+^ mononuclear phagocytes were observed in the spleens of infected animals, regardless of exposure to propofol (left panel). However, at 72 hours post-infection, propofol-treated mice displayed proportionally fewer F4/80^+^CD80^+^MHC-II^+^ mononuclear phagocytes in the spleens compared with infected control animals. No significant changes in the proportion of neutrophils were observed (right panel). *p<0.05, ***p<0.001.

Based on the changes in serum cytokine and chemokine profiles of animals sedated with propofol, particularly the significant increase in the monocyte recruitment chemokine MCP-1 (CCL2) and the neutrophil chemoattractant KC (CXCL1) and the early increase in bacterial burdens at 96 hours, we determined if drug treatment altered the presence of innate immune effector cells at sites of infection. While splenic architecture was maintained in animals infected with *Lm* in the absence of propofol and in animals exposed to propofol in the absence of infection (Figure D in S1 File), the spleens of animals that were both sedated and infected exhibited significant levels of fibrosis as well as dissolution of spleen structure (Figure D in S1 File). The spleens of these animals were also significantly smaller in size (Fig 4A), contained fewer total spleen cells (Fig 4B), and exhibited evident areas of cell necrosis (Fig 4A).

**Fig 5:**
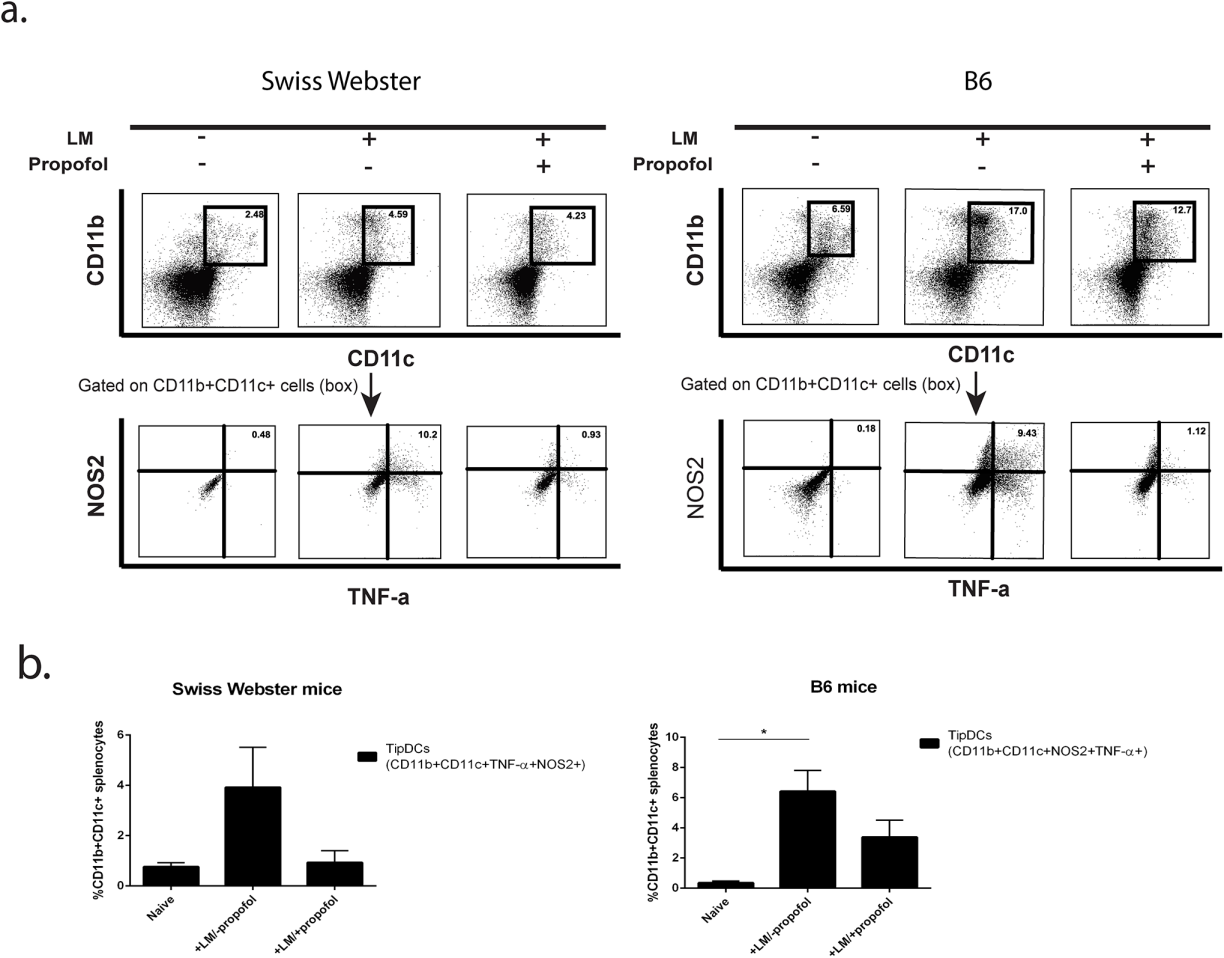
Propofol treatment decreases the number of TNF-α and iNOS-producing DCs in spleens of *Lm*-infected mice. (a) FACS analysis of cell populations expressing CD11b and CD11c on the cell surface that produce both TNF-α and iNOS in the spleens of *Lm* infected animals in the presence and absence of propofol. Experiments were done in parallel with Swiss Webster mice (left panels) and C57/Bl6 mice (right panels) to show that similar trends could be detected in both inbred and outbred strains. (b). Graphical representation of (a). All FACS experiments are representative data from at least 3 independent experiments with 4–5 animals per treatment group per time point. Error bars indicate data ± SEM. While a trend was observed for reduced numbers of TipDCs in the spleens of animals exposed to propofol, this trend did not reach statistical significance.

Propofol did not reduce the recruitment of CCR2^+^Ly-6C^+^ mononuclear phagocytes to the spleen following bacterial infection or alter the number of differentiated F4/80^+^CD80^+^MHC-II^+^ mononuclear phagocytes at 24 hours post-infection (Fig 4D). In contrast, while the total numbers of spleen cells were reduced at 72 hours post-infection, including CCR2^+^Ly6C^+^ (monocytes), CD11c^+^ MHC Class II^+^ (dendritic cells), and CD11b^+^Ly6G^+^ (neutrophils), the relative proportions of these cells stayed similar to those observed in control animals while F4/80^+^CD80^+^MHC-II^+^ mononuclear phagocytes were disproportionally decreased relative to other cell populations (Fig 4B and 4C). Mice treated with propofol in the absence of infection did not show significant alterations in numbers of F4/80^+^CD80^+^MHC-II^+^ mononuclear phagocytes (Figure E in S1 File), nor did propofol affect differentiation of primary bone marrow cells into macrophages (Figure E in S1 File). One additional myeloid cell type, the inflammatory monocyte-derived TNF and iNOS-producing dendritic cells (TipDCs), have been shown to play a key role in host resistance to *Lm* infection through direct microbial killing as well as through coordination of immune responses [28]. Mice exposed to propofol had a noticeable trend, though not statistically significant, towards reduced TipDC populations (Fig 5A and 5B), both in Swiss Webster mice and in C57/Bl6 mice, where this cell population was originally defined [28]. Propofol exposure thus reduces total innate immune effector cells at sites of infection with an increased proportional reduction in F4/80^+^CD80^+^MHC-II^+^ mononuclear phagocytes and a trend towards reduced TipDC populations.

### Propofol exposure reduces both splenic marginal zone macrophages and T cells

**Fig 6:**
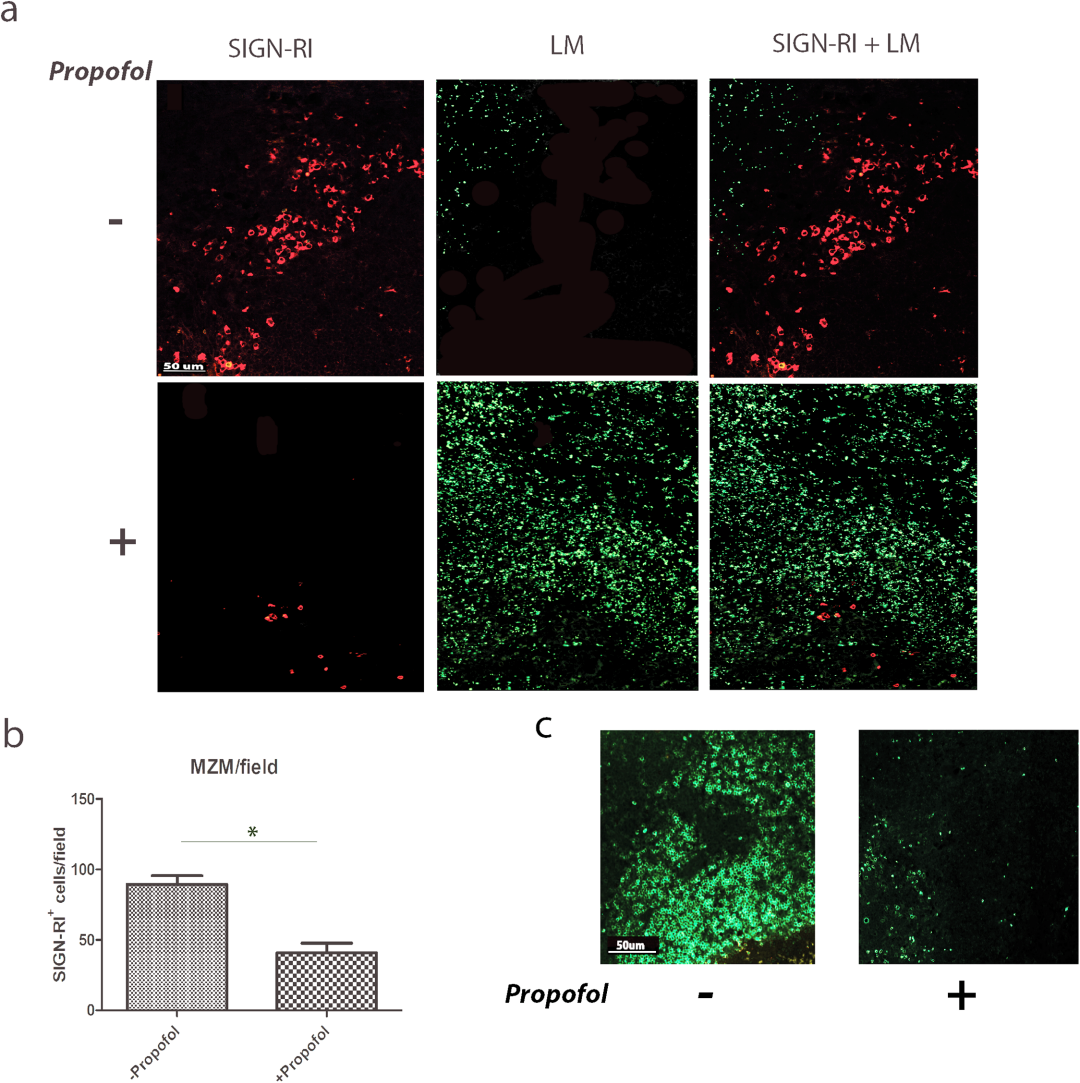
Propofol exposure leads to reduced numbers of marginal zone macrophages and T cell populations in the spleen, and facilitates dissemination of *Lm* into the white pulp. Mice were infected with 2 x 10^4^ CFU *Lm* via intravenous tail vein inoculation in the absence or presence of propofol and sacrificed at 72 hours post-infection. Spleens were harvested, fixed, and antibody-stained for the marginal zone macrophage marker SIGN-RI (red) as well as *Lm* (green) (a) or for the pan-T cell marker CD3 (green) (c). Images shown are all taken at a 10x magnification and are representative of 3 independent experiments. (b). Quantitation of marginal zone macrophages in the presence or absence of propofol in spleens of *Lm*-infected animals. Data is an average of 2–4 animals per treatment group, with counts taken from 5 fields per spleen section. Error bars indicate data ± SEM. * p<0.05.

Macrophages of the splenic marginal zone (marginal zone macrophages, or MZMs) are important for trapping blood borne particulate antigens and pathogens [7]. *Lm* entering the spleen from the blood are taken up by MZMs to prevent bacterial dissemination into the white pulp [29,30]. Consistent with the data obtained via flow cytometry with the spleens of infected mice, propofol sedated mice infected with *Lm* displayed reduced numbers of MZMs in the spleen at 3 days post-infection in comparison to vehicle-treated controls (Fig 6A and 6B), leading to bacterial dissemination throughout the white pulp (Fig 6A bottom panels) and resulting in an apparent reduction in T cell populations (Fig 6C).

### Propofol anesthesia increases host susceptibility to *Staphylococcus aureus* infection

**Fig 7:**
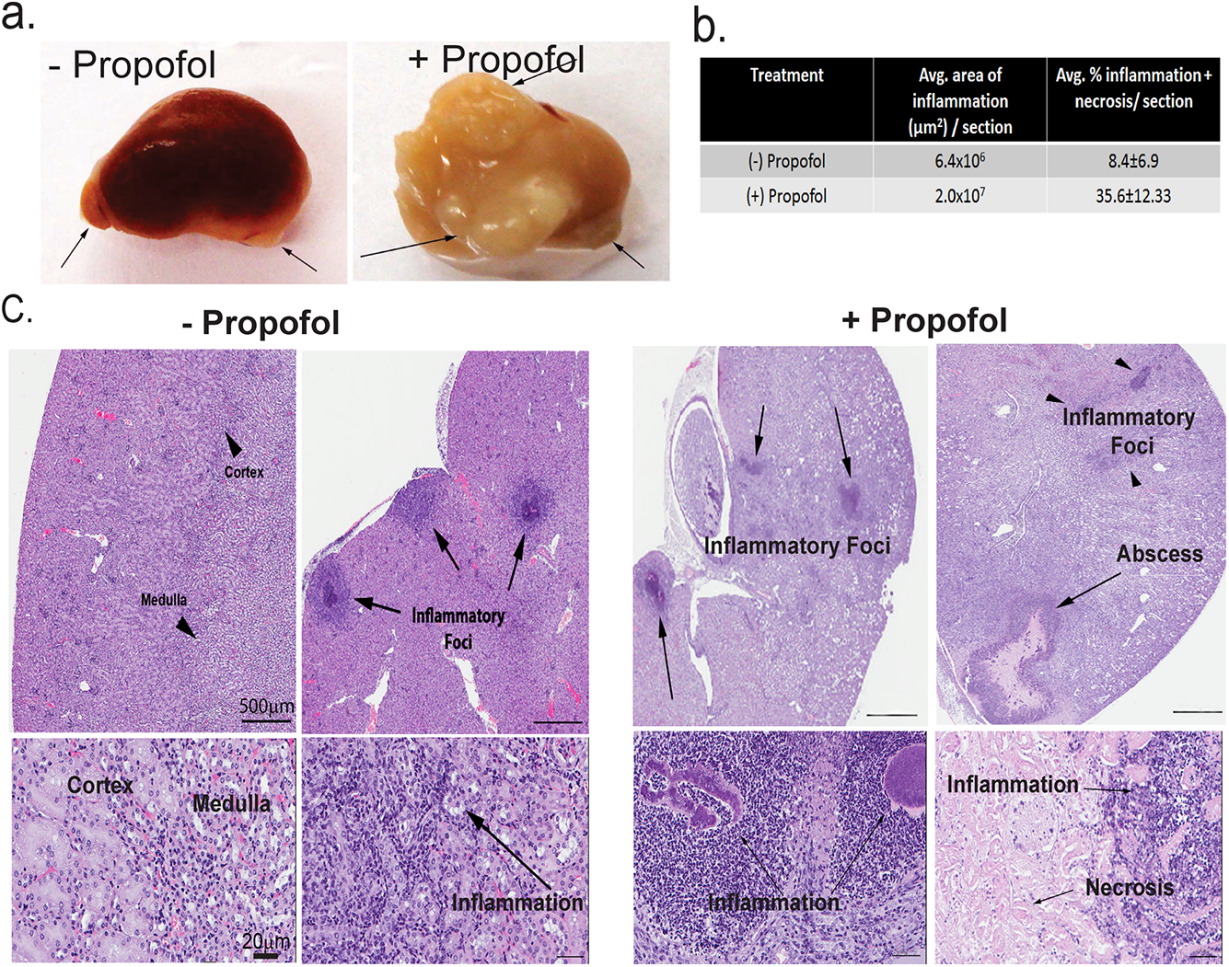
Propofol increases host susceptibility to *S*. *aureus* infection. (a). Mice were intravenously infected with 1 x 10^6^ CFU *S*. *aureus* USA300 ± propofol and kidneys were isolated at 14 days post-infection. Arrows: abscess formation. (b). Levels of inflammation and necrosis in the kidney expressed as percentage of histological sections ± SEM, with 5–6 animals per group. (c). Mice were infected with 1 x 10^6^ CFU *S*. *aureus* USA300 ± propofol and sacrificed at 7 days post-infection. Kidneys were fixed and processed for H&E staining. Propofol increased inflammation, caused the loss of kidney compartmentalization, and increased the number and size of abscesses. Left: images from 2 mice infected with *S*. *aureus* USA300 without propofol. Right: images from 2 mice infected with *S*. *aureus* USA300 with propofol. Top: 2x magnification; bottom: 20x magnification.

To determine if propofol increases host susceptibility to pathogens other than *Lm*, mice were intravenously sedated with propofol and intravenously infected with methicillin-resistant *S*. *aureus* USA300, the strain responsible for the majority of community-acquired MRSA infections [31]. Propofol treatment enhanced the susceptibility of intravenously infected mice to *S*. *aureus* both in terms of increased bacterial burdens in target organs (Figure F in S1 File) and in the total area of the kidney affected by inflammation and/or abscess formation (Fig 7A–7C). Propofol thus increases host susceptibility to at least two important pathogens that occupy very distinct replication niches within an infected host.

## Discussion

Our results indicate that while the sedative effects of propofol are short acting, brief sedation lasting less than five minutes at physiologically relevant concentrations was sufficient to significantly increase host susceptibility to both intravenous *Lm* and *S*. *aureus* infection. The effects of propofol on host immunity extended beyond the brief periods of sedation as mice that were sedated and allowed to recover for up to four days prior to infection continued to exhibit increased bacterial replication in target organs following intravenous administration of bacteria. Propofol is a highly lipophilic drug, and once introduced into the bloodstream it becomes quickly distributed into tissues [32]. The drug is then rapidly modified in the liver by Cytochrome P450 2A6 enzymes, at which point metabolites enter the bloodstream and can persist within a variety of tissues for up to several days [33]. Given that very brief periods of propofol sedation had relatively long lasting effects on host susceptibility to infection, we speculate that metabolites derived from propofol are likely responsible for the observed alterations in host immune responses that continue to occur long after the recovery from sedation.

In comparison to the alternative anesthetics pentobarbital and ketamine, propofol was unique with respect to its ability to increase host susceptibility to infection (Fig 2). Propofol and pentobarbital both bind to the GABA-A receptor to induce anesthesia, and GABA-A receptors are expressed on a variety of immune cells, including macrophages and T cells [25,34]. Changes in GABA-A receptor expression or activation patterns have been previously associated with immunomodulation [35]. The results presented here, however, suggest that the overall effects of propofol on immune clearance may be independent of GABA-A receptor binding given that pentobarbital did not similarly influence host susceptibility to microbial infection. We speculate that a propofol metabolite may target an alternative receptor that leads to changes in host immune signaling and impedes the recruitment and/or activity of immune effector cells at sites of infection.

**Fig 8:**
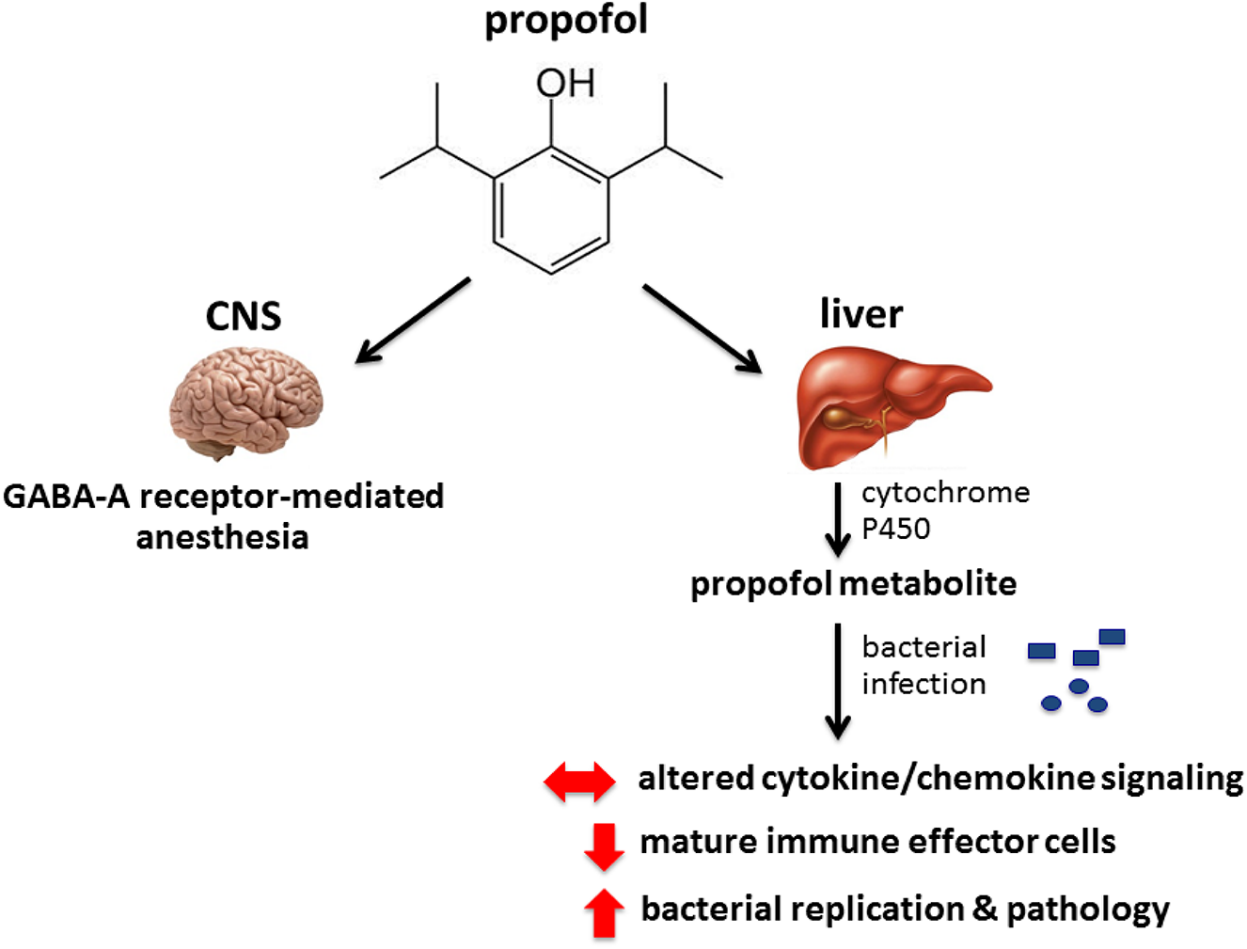
Model of the effects of propofol on host susceptibility to microbial infection. Propofol binds to the GABA-A receptor in the CNS to induce anesthesia. Upon infection, propofol increases bacterial translocation across physiological barriers and inhibits immune clearance of bacteria from target organs. Propofol is speculated to mediate immunosuppression through the action of metabolic intermediates produced in the liver that may bind to alternative receptors and alter patterns of cytokine and chemokine signaling in response to infection. Propofol sedation inhibits the recruitment and/or activity of immune effector cells at sites of infection, thereby increasing bacterial replication and organ pathology.

Monocytes and TipDCs may both be derived from CCR2^+^ myeloid progenitors [36] and play pivotal roles in limiting *Lm* infection [37]. Interestingly, high expression levels of the monocyte chemoattractant protein MCP-1 (CCL2) have been associated with defects in the recruitment of monocytes to sites of infection and with subsequent increases in host susceptibility to *Lm* [15,36,38,39]. While propofol exposure increased serum titers of MCP-1 (CCL2) to levels similar to those associated with monocyte recruitment defects (Fig 3A), there was no appreciable difference in the numbers of CCR2^+^Ly-6c^hi^ monocytes recruited to the spleen at 24 hours following *Lm* infection irrespective of drug treatment. However, propofol sedation significantly reduced the total numbers of spleen cells by 72 hours post-infection and was associated with significant necrosis suggesting the resident and recruited innate immune cells were not sufficiently functional to contain the infection. Propofol exposure was observed to disproportionally affect the frequency of F4/80^+^CD80^+^MHC-II^+^ mononuclear phagocytes present at sites of infection, along with evidence of a trend in TipDC reduction (Figs 4 & 5). Differentiation of monocytes into TipDCs and F4/80^+^CD80^+^MHC-II^+^ mononuclear phagocytes is dependent on the presence of IFN-γ [40], the levels of which were initially high in propofol-treated animals and then dropped to levels similar to those observed in non-sedated control animals (Fig 3A). These results appear consistent with a model in which propofol exposure affects the recruitment, and/or activation or differentiation of innate immune effector cells at sites of infection. These effects may be through alteration of cytokine signaling pathways and may be subsequent to the initial CCR2^+^ monocyte trafficking to sites of infection (Fig 8).

Most notably, propofol exposure dramatically reduced populations of MZMs in the spleen, and this reduction was accompanied by extensive dissemination of *Lm* into the white pulp (Fig 6). MZMs in the spleen are known to rapidly phagocytose *Lm* present in blood [8,29], thereby reducing bacterial dissemination into the splenic white pulp compartments consisting primarily of T cells and other lymphocytes [16] that require interaction with antigen presenting cells for activation [30]. The reduction in MZMs would mean fewer cells capable of *Lm* phagocytosis, degradation, and antigen presentation for the activation of T cells, a process that occurs as early as 48 hours following intravenous infection with *Lm* [30]. Dissemination of large numbers of *Lm* into the white pulp may also account for the apparent reduction in T cell populations observed following propofol exposure (Fig 6C) as *Lm* has been shown to induce apoptosis in T lymphocytes [41], which in turn has been implicated in the suppression of host inflammatory responses.

Significantly, exposure to propofol dramatically increased host susceptibility to infection with methicillin-resistant *S*. *aureus* strain USA300, the current epidemic strain of community-acquired MRSA infections (Fig 7 and Figure F in S1 File) [31]. Given that *S*. *aureus* and *Lm* occupy distinct host niches and activate different immune defense programs [42–44], propofol thus appears to broadly increase host susceptibility to infectious pathogens. Exposure to propofol resulted in gross changes in organ pathology in the spleen and kidney during *Lm* and *S*. *aureus* infection, respectively (Figs 4 & 7). While the disparity in bacterial burdens between infected controls and propofol-treated animals was far greater for animals infected with *Lm* versus *S*. *aureus*, enhanced host susceptibility to *S*. *aureus* infection was strikingly evident from the significant increase in abscess formation and necrosis that occurred in the kidneys of propofol-treated animals (Fig 7A–7C). Taken together, these results demonstrate that propofol, or metabolites derived from propofol, broadly influence host immune responses so as to promote pathogen replication and exacerbate tissue damage. Alterations in immune cell populations induced by propofol are not evident in the absence of infection, suggesting that propofol influences signaling cascades initiated in response to pathogen invasion.

Propofol is one of the most commonly used anesthetic induction agents in hospitals across the United States [45], and often patients are sedated for hours or even days in the case of the critically ill in the ICU [46]. Propofol has become the anesthetic induction agent of choice for many in-patient and out-patient procedures, with the result that large number of patients are exposed to the drug for both brief and extended periods of time [47–50]. This study illustrates the significant impact of brief periods of propofol exposure on host immunity using well-established *in vivo* infection models. The potential impact of these findings on the selection on anesthetic induction agents for human patients merits further investigation.

## Supporting Information

S1 File Additional figures for ‘Propofol Increases Host Susceptibility to Microbial Infection by Reducing Subpopulations of Mature Immune Effector Cells at Sites of Infection’. (PDF)
