# Supplementary material for: Propofol Increases Host Susceptibility to Microbial Infection by Reducing Subpopulations of Mature Immune Effector Cells at Sites of Infection
Source: PLoS One. 2015 Sep 18;10(9):e0138043. doi: 10.1371/journal.pone.0138043 (PMC4575148; doi:10.1371/journal.pone.0138043)
Supplement: S1 File — (PDF) [file pone.0138043.s001.pdf]

S1 File

SUPPLEMENTARY MATERIALS:

Fig. A. Propofol does not affect *Lm* viability, nor does it enhance *Lm* intracellular growth in primary macrophages.

Fig. B. Propofol increases the efficiency of *Lm* translocation across the blood-brain barrier.

Fig. C. The immunosuppressive effect of propofol is diminished for infections established 7 days post-sedation.

Fig. D. Exposure to propofol increases spleen pathology during *Lm* infection but does not affect spleen architecture in the absence of infection.

Fig. E. Propofol alone in the absence of *Lm* infection does not alter mononuclear phagocyte numbers in mouse spleens nor does it alter differentiation of primary bone marrow cells into macrophages.

Fig. F. Propofol increases bacterial burdens at 7 and 14 days post-infection in animals infected with methicillin-resistant *S. aureus*.

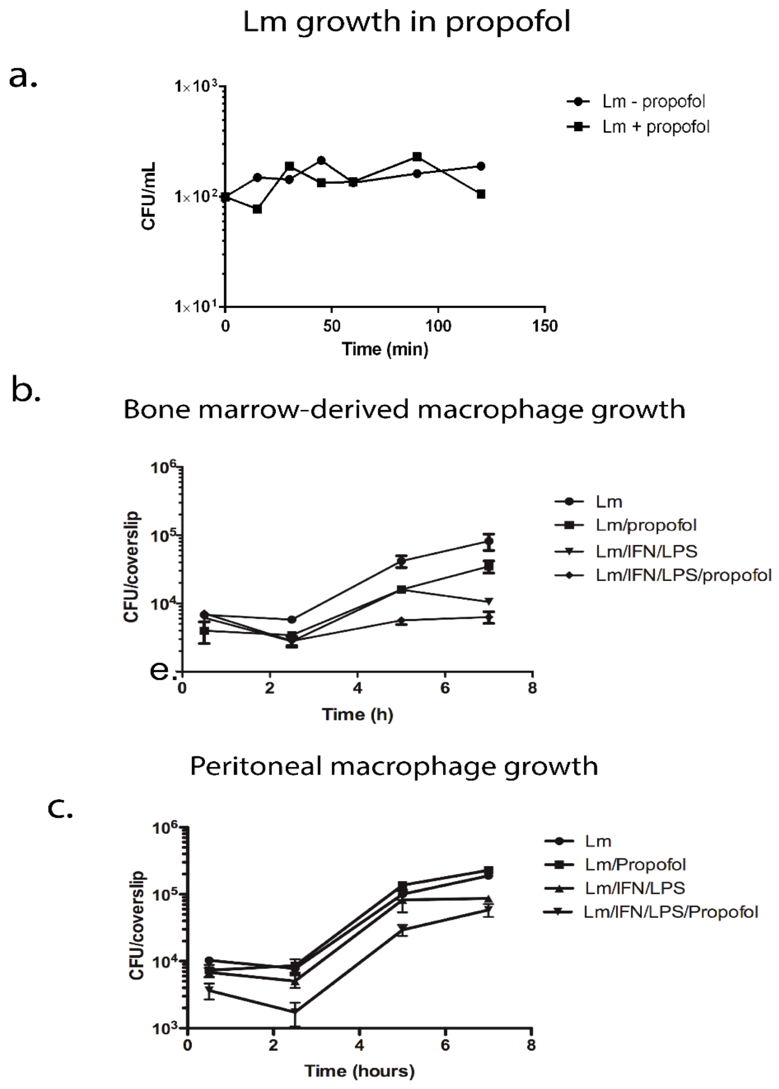

Fig. A. Propofol does not affect *Lm* viability, nor does it enhance *Lm* intracellular growth in primary macrophages. (a) *Lm* cultures incubated in propofol or intralipid carrier maintain viability. Overnight cultures of *Lm* were grown in BHI broth, diluted 1:20 in 10mL fresh BHI broth and grown to mid-log phase (OD 0.6). Bacteria were washed and re-suspended in PBS at a concentration of 2 x 10^4 CFU/100µL, the same concentration that was used for mouse infections. 0.5mL of bacterial PBS suspension was mixed 1:1 with 0.375mg/mL propofol suspension or Intralipid vehicle solution and aliquots were diluted and plated at the indicated time points for CFU enumeration. (b,c) Propofol does not enhance the growth of *Lm* in primary macrophages. Primary bone marrow cells (b) or peritoneal macrophages (c) were isolated from female Swiss-Webster mice. Macrophages were cultured in tissue culture dishes containing glass coverslips and infected with *Lm* at an MOI of 0.1. After 30 minutes of infection, the coverslips were washed three times with 37°C PBS, and prewarmed media containing 10 ug/ml gentamicin was added. Coverslips were removed at the indicated time points, macrophages were lysed in sterile water with vortexing, and dilutions of the cell lysates were plated for viable CFU. Data shown is representative of 3 independent experiments.

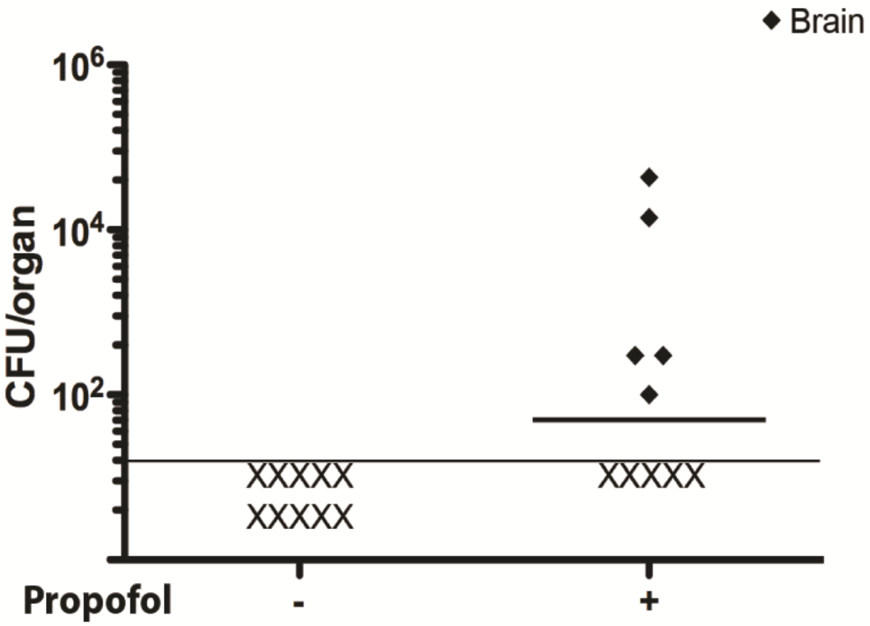

Fig. B. Propofol increases the efficiency of *Lm* translocation across the blood-brain barrier. Mice were intravenously infected via tail vein with a low dose (2 x 10^3 CFU) of *Lm* in the presence or absence of propofol. At three days post-infection the animals were euthanized, and bacterial burdens determined in the brain. Data shown is representative of two independent experiments.

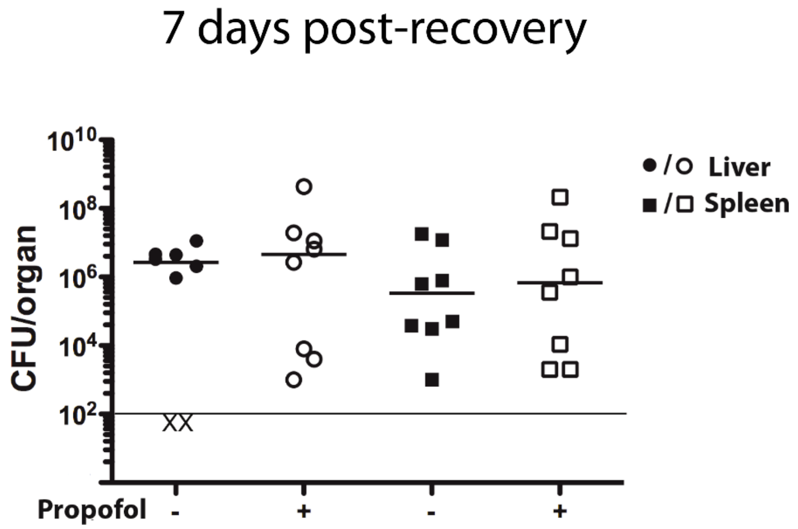

Fig. C. The immunosuppressive effect of propofol is diminished for infections occurring 7 days post-sedation. Mice were briefly anesthetized with 18.75 mg/kg propofol (approximately five minutes of sedation) and allowed to recover for 7 days before intravenous infection with 2 x 10^3 CFU of *Lm*. Animals were euthanized at three days post-infection and bacterial burdens were determined in the liver and spleen. Data shown is representative of two experiments.

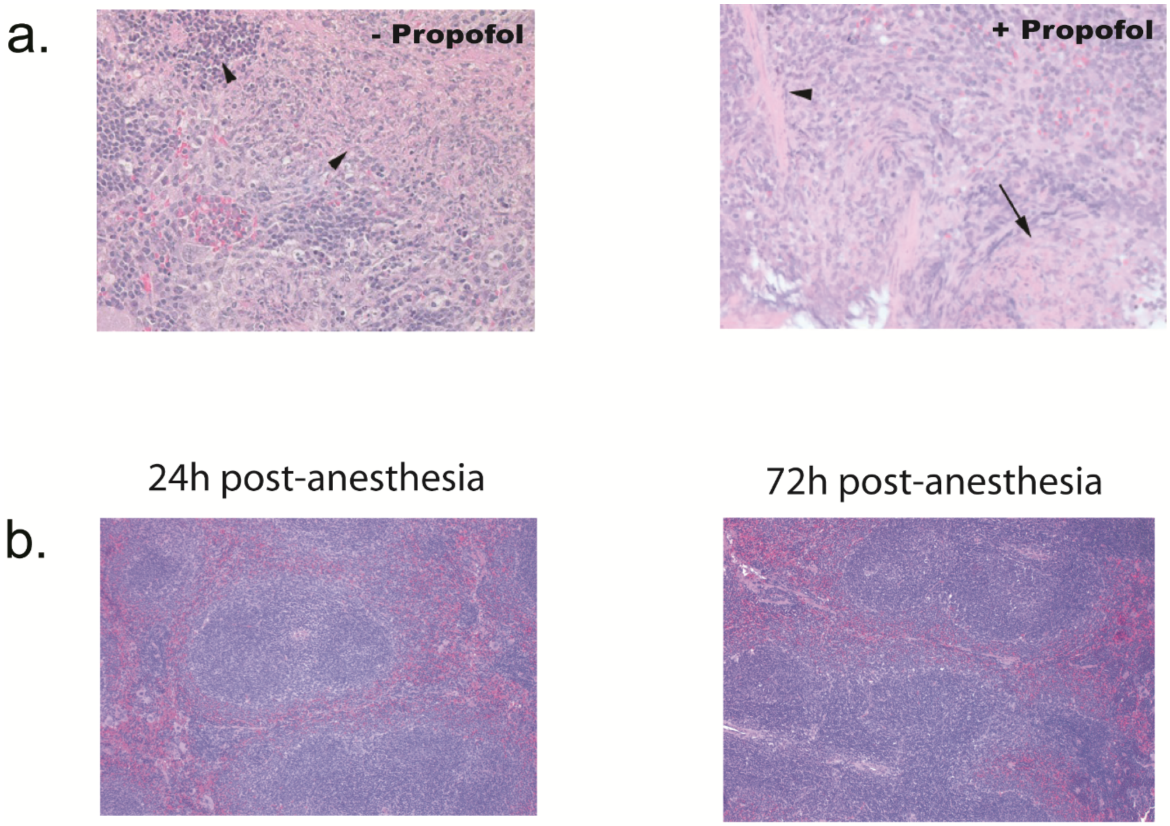

Fig. D. Propofol anesthesia does not affect spleen architecture in the absence of infection. (a). Propofol-treated mice exhibit a loss of splenic architecture. Mice were intravenously infected with 2 x 10^4 CFU of *Lm* in the presence and absence of propofol. Spleens were isolated at 72 hours post-infection, fixed, and stained with hematoxylin and eosin staining. Arrowheads, left panel: compartmentalization of red and white pulp. Arrowhead, right panel: fibroid deposit; arrow: necrosis. (b). Animals were injected with 18.75 mg/kg propofol via tail vein and sacrificed 24 or 72 hours post-sedation. Spleens were isolated and processed for staining. Representative image from 3 animals per treatment group.

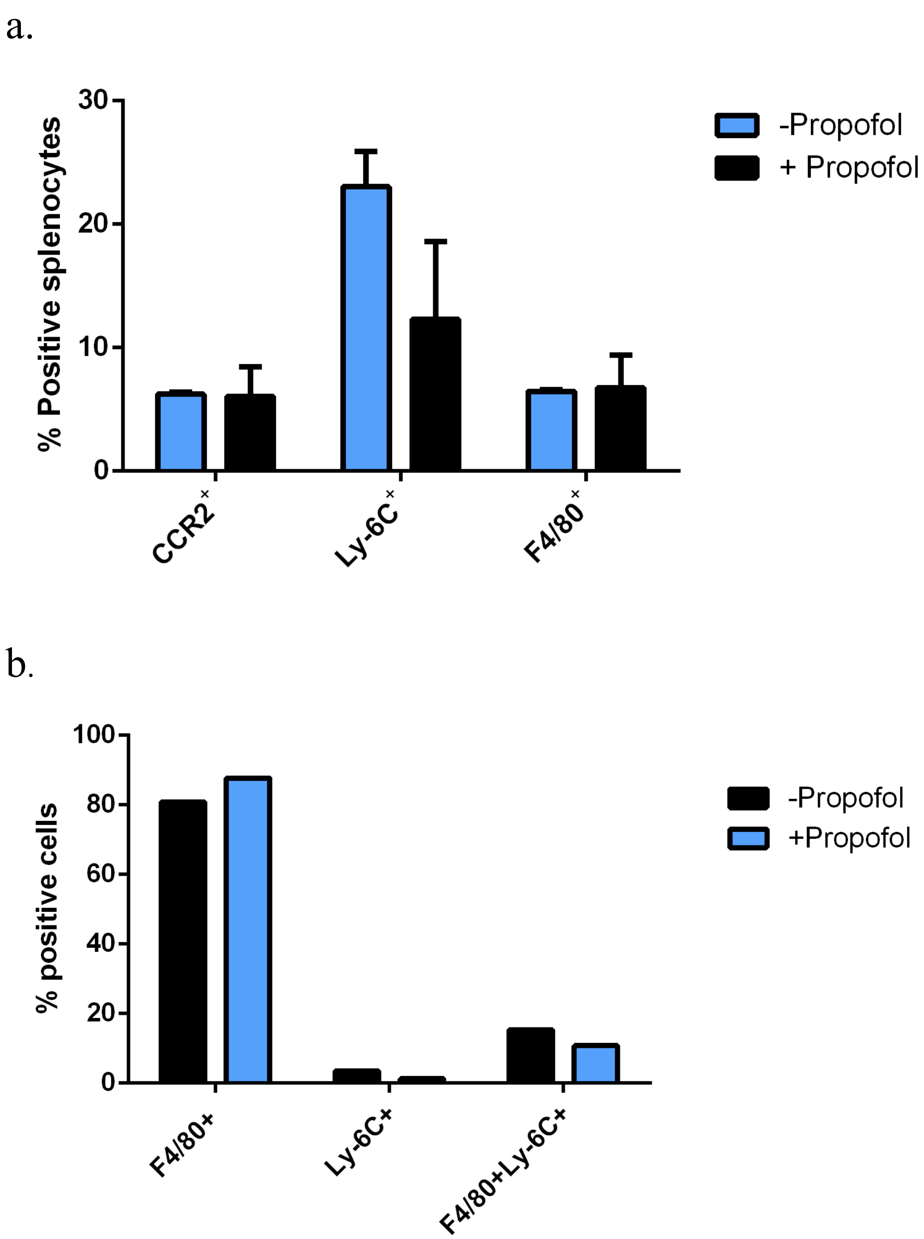

Fig. E. Propofol alone in the absence of *Lm* infection does not alter mononuclear phagocyte numbers in mouse spleens nor does it alter differentiation of primary bone marrow cells into macrophages. (a). Mice were injected with vehicle solution or propofol by tail vein injection and sacrificed 72 hours post-injection. Spleens were isolated and processed for FACS analysis. No significant differences in $CCR2^+$ inflammatory monocytes, Ly-$6C^+$ monocytes, or $F4/80^+CD80^+MHC$-II^+ mononuclear phagocytes were seen between treatment groups. Graph representative of 2-4 animals per treatment group. (b). Primary bone marrow cells were isolated as described in *Materials and Methods*. Cells were exposed to Intralipid vehicle solution, propofol, or no additive in culture media for 6 days until fully differentiated. Cells were isolated and processed for FACS analysis. Exposure to propofol did not alter numbers of $F4/80^+$ macrophages, Ly-$6C^+$ monocytes, or $F4/80^+$Ly-$6C^+$ mononuclear phagocytes. Representative data from two independent experiments.

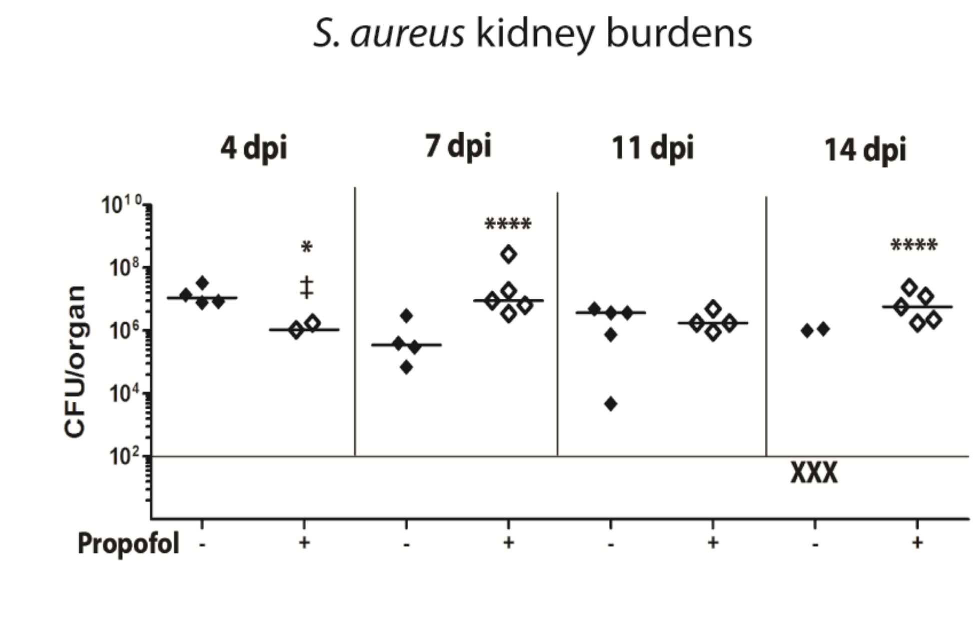

Fig. F. Propofol increases bacterial burdens at 7 and 14 days post-infection in animals infected with methicillin-resistant *S. aureus*. Mice were infected with 1 x 10^6 CFU *S. aureus* USA300 ± propofol. Animals were sacrificed and kidneys isolated to assess bacterial burdens at the indicated time points. Data is representative from 2 independent experiments with at least 3 animals per time point per treatment group. * $p<0.05$, **** $p<0.0001$.
